# FlexPES: a versatile soft X-ray beamline at MAX IV Laboratory

**DOI:** 10.1107/S1600577523003429

**Published:** 2023-05-09

**Authors:** Alexei Preobrajenski, Alexander Generalov, Gunnar Öhrwall, Maxim Tchaplyguine, Hamed Tarawneh, Stephan Appelfeller, Eleanor Frampton, Noelle Walsh

**Affiliations:** a MAX IV Laboratory, Lund University, Box 118, Lund 221 00, Sweden; ESRF – The European Synchrotron, France

**Keywords:** photoelectron spectroscopy, NEXAFS, multi-coincidence, soft X-rays

## Abstract

FlexPES is a soft X-ray beamline on the 1.5 GeV storage ring at MAX IV Laboratory, Sweden, providing horizontally polarized radiation in the 40–1500 eV photon energy range and specializing in high-resolution photoelectron spectroscopy, fast X-ray absorption spectroscopy and electron–ion/ion–ion coincidence techniques. The beamline is split into two branches currently serving three endstations, with a possibility of adding a fourth station at a free port. The refocusing optics provides two focal points on each branch, and enables either focused or defocused beam on the sample. The endstation EA01 at branch A (Surface and Materials Science) is dedicated to surface- and materials-science experiments on solid samples at ultra-high vacuum. It is well suited not only to all flavours of photoelectron spectroscopy but also to fast (down to sub-minute) high-resolution X-ray absorption measurements with various detectors. Branch B (Low-Density Matter Science) has the possibility to study gas-phase/liquid samples at elevated pressures. The first endstation of this branch, EB01, is a mobile setup for various ion–ion and electron–ion coincidence techniques. It houses a versatile reaction microscope, which can be used for experiments during single-bunch or multi-bunch delivery. The second endstation, EB02, is based on a rotatable chamber with an electron spectrometer for photoelectron spectroscopy studies on primarily volatile targets, and a number of peripheral setups for sample delivery, such as molecular/cluster beams, metal/semiconductor nanoparticle beams and liquid jets. This station can also be used for non-UHV photoemission studies on solid samples. In this paper, the optical layout and the present performance of the beamline and all its endstations are reported.

## Abstract 2

A new versatile beamline for experiments with soft X-ray absorption, photoelectron emission and electron–ion coincidence spectroscopies is available at MAX IV Laboratory, Sweden, serving user communities from ultra-high-vacuum surface science to materials science and low-density matter research.

## 1. Introduction

FlexPES is a soft X-ray (40–1500 eV) beamline at the 1.5 GeV storage ring of the MAX IV Laboratory, with a focus on photoelectron spectroscopy (PES), X-ray absorption spectroscopy (XAS) and electron–ion coincidence techniques. It has been designed as a general-purpose soft X-ray beamline featuring up to four endstations with different sample environments and beam focusing conditions, capable of attracting diverse user communities and adapting quickly to cater to evolving user demands.

The broad spectrum of research areas covered by the facilities at FlexPES includes catalysis, solar cells, heterojunctions for electronics, novel 2D materials, sensor materials, novel batteries, corrosion and protective coatings, environment- and climate-relevant aerosols, molecular/cluster reactions, *etc*. The chemistry of various sample transformations can be understood by following the PES and XAS spectral shape as a function of experimental conditions, from on-surface reactions on atomically clean flat substrates to photo-induced dynamics and fragmentation in molecules and free clusters.

The construction of FlexPES was funded by the Swedish Research Council in 2014 as a part of the Transfer Package Project upon the transition from the predecessor MAX-lab to the present MAX IV, which was based on re-using selected key components from several MAX-lab beamlines (for economic reasons). In particular, the X-ray source, the monochromator and two of the endstations were transferred from various beamlines at MAX-lab and integrated into the new design after refurbishing. By now, all design goals of the project have been achieved, and the baseline scope of the planned instruments and techniques was delivered already by 2020.

The beamline is split into two branch lines serving presently three endstations, with a possibility of adding a fourth station at the reserved open port. The optics are similar for both branch lines, providing either focused or defocused beam on the sample. The Surface and Material Science (SMS) branch hosts essentially experiments on solid surfaces at ultra-high vacuum (UHV) conditions, while the Low-Density Matter (LDM) branch is mainly intended for experiments on volatile targets at somewhat higher pressures.

The first (permanent) endstation EA01 (where ‘E’ stands for ‘endstation’, ‘A’ is the A-/SMS- branch, ‘01’ means the first station from the ring) offers high-resolution ultraviolet PES (UPS), core-level X-ray PES (XPS) and near-edge X-ray absorption fine-structure (NEXAFS) spectroscopy in several detection modes. While targeting mainly core-level studies, the station has capabilities for measuring valence band structures with angle-resolved photoemission (ARPES). This setup is a deeply refurbished surface science endstation transferred from the D1011 beamline at MAX-lab; it has been in user operation since February 2020.

The second (also permanent) endstation EB02 (B- or LDM-branch) is focused primarily on PES studies in liquid, molecular and free cluster jets, but can also be used for investigations of solid samples, especially non-UHV compatible ones. The station enables a rapid mounting of a variety of different sample delivery systems/jet sources belonging to the MAX IV LDM community. This station was also transferred from the MAX II ring at MAX-lab (beamline I411); it is now upgraded and has been in user operation since February 2020.

The third (mobile) endstation EB01 or ICE (‘Ions in Coincidence with Electrons’) features a reaction microscope (REMI) for multi-coincidence experiments on volatile targets. This is a new station designed to be compatible with a number of MAX IV LDM-relevant beamlines. The first general user experiments were performed with the ICE REMI in the autumn of 2022.

## 2. Beamline overview

### 2.1. X-ray source

The MAX IV 1.5 GeV electron storage ring is based on a compact double-bend achromat lattice for the production of bright soft X-ray and UV radiation. With its circumference of 96 m and 12 achromat sections, it is using the same magnet technology as the MAX IV 3 GeV ring, providing very low emittance of the electron beam (Eriksson *et al.*, 2013 ▸). This is an excellent prerequisite for designing beamlines with small and bright X-ray sources. At the centre of the FlexPES straight section, the root-mean-square (RMS) values of the electron beam size and divergence are 184 µm (h) × 13 µm (v) and 33 µrad (h) × 5 µrad (v), respectively. The 1.5 GeV ring operates today with a current of 500 mA and a top-up period of 10 min.

**Figure 1:**
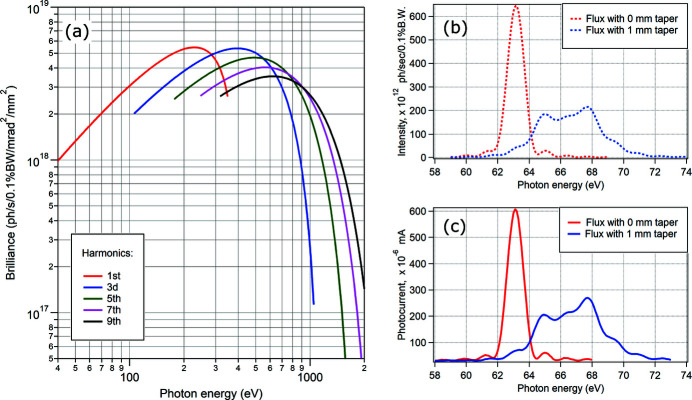
(*a*) Calculated brilliance distribution of synchrotron radiation from the FlexPES LPU with 500 mA in the ring and 0.1% bandwidth of the monochromator. (*b*) Calculated undulator spectra (first harmonic) for the gap of 20 mm and the acceptance cone of 100 µrad × 100 µrad in the case of straight (red) and tapered by 1 mm (blue) magnet arrangement. (*c*) Same as (*b*) but measured experimentally with a photodiode after the exit slit.

The linearly polarizing undulator (LPU) U54.4 from the beamline I311 at MAX-lab is now used as the X-ray source for FlexPES. It has 49 periods, period length of 54.4 mm, effective vertical field of 0.85 T and effective *K*-value of 4.315. With its minimal gap of 16 mm, it can deliver photon energies starting from 38 eV, while the practical limitation set by the monochromator is around 42 eV. This LPU can also be tapered for broadening the undulator harmonics. This device had new magnets installed in 2010 and its magnetic structure has been fully characterized prior to its installation at FlexPES. It was also equipped with new motors, encoders and sensors, and integrated into the general control system of MAX IV. Fig. 1 ▸(*a*) shows the brilliance of this source in a straight section of the MAX IV 1.5 GeV ring for the five harmonics used in the entire photon energy range of the beamline. The selection of the correct harmonic (*i.e.* the proper LPU gap) is dictated by flux maximization and happens automatically depending on the monochromator settings and desired photon energy. Most typical energies for changing the LPU harmonics are 330 eV (1-to-3), 620 eV (3-to-5), 920 eV (5-to-7) and 1120 eV (7-to-9). The performance of the insertion device was characterized after the beamline alignment and energy calibration by measuring the undulator spectra and comparing them with the results calculated using the *SPECTRA* (Tanaka & Kitamura, 2001 ▸) and *XRT* (Klementiev & Chernikov, 2014 ▸) software. The undulator spectra were recorded with different energies, harmonic numbers, beamline acceptance and tapering conditions, all showing good correspondence between the measured and the calculated spectral shapes. As an example, Fig. 1 ▸ provides a comparison of the predicted (*b*) and measured (*c*) undulator spectra of the first harmonic for the on-axis radiation conditions and a gap of 20 mm.

### 2.2. Beamline optics

**Figure 2:**
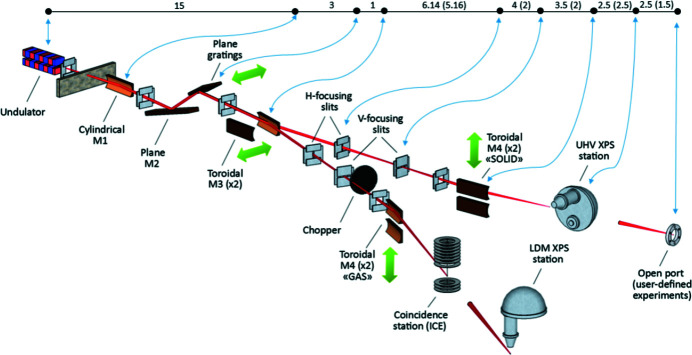
FlexPES beamline layout depicting optical elements. Distances from the X-ray source point are given on top in meters; values without (in) parenthesis correspond to the SMS (LDM) branch.

In the front-end, a water-cooled fixed mask is installed followed by a pair of water-cooled movable masks used for the beam shaping. After the front-end, the LPU radiation is deflected horizontally (by 4°) and collimated vertically by a water-cooled cylindrical mirror M1 (see a schematic of the beamline layout in Fig. 2 ▸). The size of its optical area defines the ultimate beamline acceptance of 0.62 mrad (h) × 1.36 mrad (v). In order to facilitate the heat load handling, this acceptance is normally reduced using the front-end masks; however, the irradiated footprint on M1 remains sufficient to accept nearly all of the desired monochromatic radiation. The acceptance can be further reduced by the water-cooled beam-defining apertures positioned in front of the monochromator to select the working part of the grating.

The refurbished Zeiss SX-700 plane-grating monochromator (PGM) operates in the collimated PGM scheme (Follath *et al.*, 1998 ▸). Within this scheme, the beam remains horizontal after the monochromator (for easier operation and exchange of endstations), the resolution is less sensitive to the slope errors on optical elements, and the exit slit is fixed in space for different *c*_ff_ values (the PGM parameter *c*_ff_ is the cosine ratio of the diffracted and incident angles). By varying the *c*_ff_ values, different modes of the PGM operation can be achieved (Follath & Senf, 1997 ▸): higher-order suppression mode (*c*_ff_ ≃ 1.5), high-resolution mode at the cost of intensity and spectral purity (*c*_ff_ ≃ 4–6), and standard operation as a compromise of the two extremes (*c*_ff_ = 2.25). The optical elements inside the monochromator are the plane mirror (M2) and the plane gratings (PG, up to three). Currently, one grating (1221 lines mm^−1^) can be used for the entire energy range, while a second one (400 lines mm^−1^) is used specifically for low photon energies (below 500 eV and especially below 150 eV) in photon-demanding experiments. The characteristics of the presently available gratings are summarized in Table 1 ▸.

A pair of toroidal mirrors M3a and M3b are rigidly mounted inside the M3 vacuum chamber, so that the working mirror can be selected by translating the whole chamber. In this way, the radiation can be focused onto the exit slits either in the SMS- or in the LDM-branch (see Fig. 2 ▸), with an angular separation of 8° between them. This intermediate focus is astigmatic on both branches, with the horizontal focus preceding the vertical one. The stronger focusing in the horizontal plane allows elongating the entrance arm on the next (refocusing) mirrors, M4a and M4b, in the same plane, thus increasing the demagnification in the horizontal direction and improving the aspect ratio of the beam spot at the sample, *i.e.* making it less elongated horizontally. The opening of all four slits can be varied continuously and reproducibly with a precision better than 1 µm, and their position along the beam can be adjusted to compensate for a misalignment and mirror shape errors. After the exit slits, both branches are equipped with gas ionization cells for monitoring beamline resolution with X-ray absorption spectra of reference gases.

Each M4 refocusing optics is manufactured as a pair of toroidal mirror stripes with different curvature placed on the same substrate. The working stripe can be selected by moving each of the M4 vacuum chambers up or down (see Fig. 2 ▸). The M4 mirrors deflect the beam horizontally and focus it on the sample: one stripe focuses the radiation into the upstream endstation and the other one into the downstream station. In this way, two different experimental setups can be accommodated on each branch without an additional re-focusing in-between. As an important bonus, this arrangement provides the possibility of either focusing or defocusing the beam on the sample, depending on the sample type and its sensitivity to radiation damage. The essential characteristics of all FlexPES mirrors are summarized in Table 2 ▸.

The dedicated diagnostic sections on each branch allow measurements of the beam profiles at M4 with YAG screens and cameras, as well as the total photon flux with photodiodes. For monitoring the intensity of incident radiation, I_0_ sections are placed at the end of each branch. Thin gold foils with an opening for the X-ray beam are used as I_0_ sensors, and their cleanliness is ensured by regular evaporation of fresh gold (clean I_0_ signals are essential for reliable NEXAFS data normalization). Other diagnostic tools are phospho­rus screens and cameras on the (1) beam-defining apertures before the PGM, (2) beam-defining apertures before M3 and M4, and (3) exit slits. Finally, the drain current from the M4 mirrors at both branches is constantly monitored.

On the LDM-branch, an X-ray chopper is installed right after the exit slit. It can be used in coincidence experiments to increase the separation between X-ray pulses during single-bunch operation of the 1.5 GeV ring. The chopper is a development of an earlier design (Plogmaker *et al.*, 2012 ▸), based on a rotating disc with slits at the disc perimeter; its main parameters are summarized in Table 3 ▸. In total there are 120 slits, out of which 12 have an extended length. The chopper is translated in and out of the beam on linear rails. The period between the X-ray pulses can be varied by adjusting this translation: either all 120 slits are illuminated or only the 12 longer ones are selected, thus extending the period by a factor of ten. A fixed horizontal slit is mounted just in front of the disc to ensure that the opening time is less than the pulse period in the single-bunch mode (320 ns) at the highest allowed rotation speed. The speed and phase of the disc rotation is controlled by a MAX IV-designed closed-loop system, which uses the ring bunch marker and the signal from a laser-photodiode setup mounted on either side of the disc, generating a pulse every time a slit passes by, as input. The chopper can thus be synchronized to the X-ray pulses, increasing its efficiency.

### 2.3. Beamline performance

The software package *XRT* (Klementiev & Chernikov, 2014 ▸) was used throughout the optical design and calculations of the expected performance. For estimation of energy resolution, flux and spot profiles, the real (measured) shapes of the optical elements and the real (measured) values of the undulator magnetic field were used. Heat bump deformations on M2 were estimated at the design stage using *COMSOL Multiphysics* (COMSOL, 2014 ▸) with the power density input calculated in *XRT*. The influence of the M2 heat bumps on the energy resolution was observed only at the lowest photon energies around 40 eV, corresponding to the steepest incident angles on M2. However, even in this case the bump-related energy broadening remained in the sub-meV range, therefore any heat bump deformations were ignored in the final ray tracing.

#### 2.3.1. Energy resolution

The energy resolution of the beamline was characterized by collecting and analysing NEXAFS spectra of several reference gases in the gas cells at both branches, with the typical gas pressure around 5 × 10^−4^ mbar. The most useful for extracting resolution figures in the photon energy range of interest are He, N_2_ and Ne, but additional characterization was performed also with the spectra of Ar, Kr and Xe.

**Figure 3:**
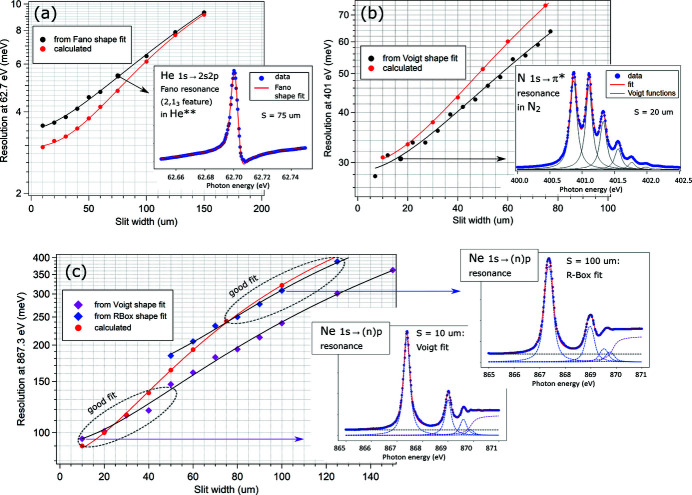
Energy resolution Δ*E* (FWHM) at the SMS branch determined from the spectra of different gases and compared with the ray-tracing calculation results obtained in *XRT*. (*a*) At 62.7 eV, with the help of the 2,1_3_ Fano resonance in He (inset: He spectrum, exit slit 75 µm), (*b*) at 401 eV, with the help of the 1*s* → 2*p*(π*) resonance in N_2_ (inset: N_2_ spectrum, exit slit 20 µm), (*c*) at 867.3 eV, with the help of the 1*s* → 3*p* transition in Ne [inset: Ne spectra taken with the slits 10 and 100 µm and fitted with Voigt profiles and rounded box (R-box) profiles, respectively]. In all cases *c*_ff_ = 2.25, and the grating line density is 1221 mm^−1^.

At lower energies, the 2,1_3_ autoionization resonance in double ionized He at 62.7 eV was used for estimating the energy resolution, which can be determined by fitting this feature with a Fano shape profile as shown in the inset of Fig. 3 ▸(*a*). The systematic fit of the spectra (*c*_ff_ = 2.25, 1221 lines mm^−1^ grating) with increasing exit slit width provides a reliable estimate of the resolution figures, with the best value of 3.5 meV, as measured at the photon energy of the resonance (62.7 eV) using a 10 µm slit. It is marginally worse than the theoretical prediction of 3.0 meV, and the discrepancy becomes even smaller with increasing slit width [see Fig. 3 ▸(*a*)].

For the middle energy range, the vibrationally resolved N 1*s* → 2*p*(π*) resonance in N_2_ at ∼401 eV is a convenient fingerprint of the resolution [see Fig. 3 ▸(*b*), inset]. We fitted all peaks by Voigt functions with the fixed Lorentzian width of 117 meV (Watanabe *et al.*, 1997 ▸), and averaged the obtained Gaussian broadening over the first four (most intense) peaks. As seen in Fig. 3 ▸(*b*), the measured resolution figures are even somewhat better than the predicted values, which may stem from a slight overestimation of the lifetime broadening used in the fit. This trend is consistent for a wide range of exit slit widths providing confidence in the fitting results.

At higher energies, the Ne 1*s* → *np* (*n* = 3, 4, 5,…) progression in Ne gas was used for estimating the beamline resolution. All individual transitions were fitted with either Voigt or rounded-box spectral shapes, and the Gaussian FWHM was obtained from the deconvolution of the 1*s* → 3*p* peak at 867.3 eV in the assumption of its Lorentzian lifetime broadening of 260 meV (Coreno *et al.*, 1999 ▸). It turned out that the Voigt shape fit works best for the smaller slit widths (below 40 µm), while the rounded-box shapes were necessary for the correct fit at larger openings (above 50 µm), as shown in Fig. 3 ▸(*c*). Also at these energies, the correspondence between the measured and the expected resolution is excellent.

The energy resolution examples shown in Fig. 3 ▸ are specific for the SMS branch, but the same analysis was performed for the LDM-branch as well. Due to shorter distances between optical elements, the same slit openings result here in slightly worse resolution and slightly higher flux, but the trends with varying energy and slit width are identical to the SMS branch trends and match the expected behaviour equally well.

In practice, dedicated software provides users with the beamline resolution figures for any arbitrary photon energy and slit opening. For PES, it also calculates the analyser-specific resolution, and the total spectral resolution.

#### 2.3.2. Energy scale and scanning

For the photon energy calibration, the offsets of optical elements in the PGM were adjusted to ensure zero energy shifts with varying *c*_ff_ values (Weiss *et al.*, 2001 ▸). After that, the energy scale was calibrated individually for each branch using the Fermi level positions measured from a clean Au sample. The accuracy and reproducibility of the energy scale is normally within 100 meV below 500 eV and somewhat worse at higher energies. For X-ray absorption spectra, the energy can be routinely scanned in the non-stop (continuous) mode involving a complex trajectory motion of the optical elements and the LPU combined with trigger-based acquisition with a number of detectors. In particular, the *c*_ff_ value can be chosen as a scan parameter of the PGM trajectory motion, thus enabling extra control over the XAS resolution and degree of higher-orders suppression in the continuous scanning mode. Typically, this mode is five to ten times faster than the stepwise mode, with the same or better spectral quality.

#### 2.3.3. Photon flux

**Figure 4:**
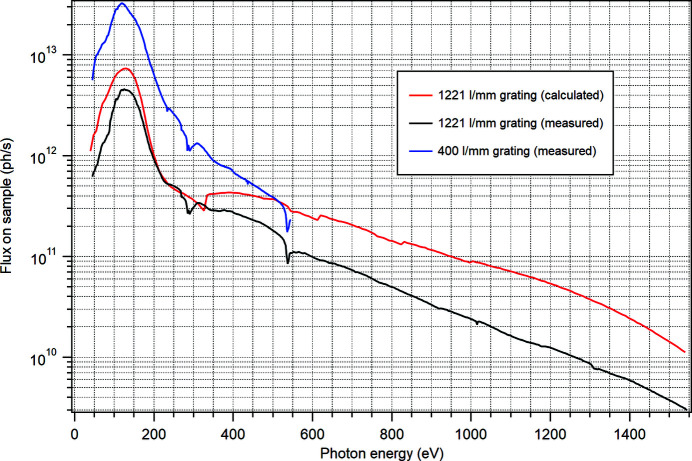
Black curve: photon flux for the entire energy range, measured at sample on the SMS branch with an exit slit of 20 µm, 1221 lines mm^−1^ grating, *c*_ff_ = 2.25 and angular beamline acceptance of 124 µrad (h) × 62 µrad (h). Red curve: ray-tracing calculation with the same parameters. Several jumps in the curve are due to the harmonic switching. Blue curve: measurement with the same parameters with the new 400 lines mm^−1^ grating.

The absolute photon flux measurements were performed with the silicon photodiode (AXUV 100 from IRD) positioned after the refocusing optics. For comparison with the calculated values, the same angular acceptance of 124 µrad (h) × 62 µrad (v) corresponding to the most typical mode of a narrow on-axis radiation cone, the 1221 lines mm^−1^ grating and *c*_ff_ = 2.25 were used both in the measurements and in the ray tracing. At FlexPES, the LPU gap motion is synchronized with the PGM motion, and the flux curves were recorded using energy scanning across the harmonics 1, 3, 5, 7 and 9 for different energy intervals. An example of the resulting flux curves in the entire energy region is shown in Fig. 4 ▸. The ‘raw’ measured curve is normalized to the responsivity of the photodiode and to the photon energy. As can be seen, the overall shape of the measured curve is very similar to the expected behaviour, with the C 1*s* and O 1*s* absorption features being absent in the calculated curve. The measured flux becomes lower than expected after the C and O absorption features, and the efficiency of the old 1221 lines mm^−1^ grating at higher photon energies is reduced, suggesting a need for replacing it with a new grating in the future. This fact is limiting the guaranteed photon energy range to 1500 eV, although technically energies up to 1600 eV can be reached. On the whole, the agreement with the ray-tracing result is very satisfactory. The flux on sample is increasing strictly linearly with the opening of the exit slit for both branches, as a sign of good alignment. The characterization of the flux and resolution with the new 400 lines mm^−1^ grating (from autumn 2022) has also been performed, with an example of the flux measurement shown in Fig. 4 ▸ (blue curve), showing a gain by a factor of five to ten in the low-energy range in comparison with the high-density grating.

#### 2.3.4. Beam spot profile

**Figure 5:**
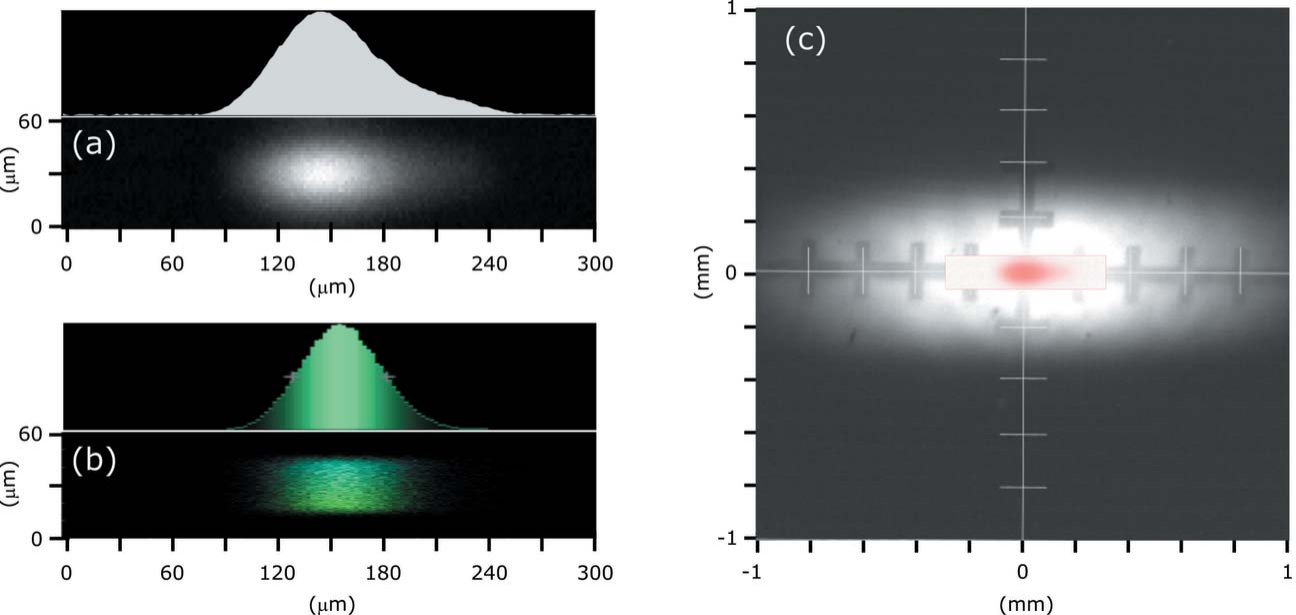
(*a*) Focused beam spot at the sample position photographed in the EA01 endstation; *E* = 265 eV, slits 300 µm (h) × 40 µm (v); its intensity distribution in the horizontal direction is shown above. (*b*) Profile of the focused beam spot at the sample position obtained from the ray tracing with the same parameters as in (*a*). (*c*) Defocused beam spot profile measured as in (*a*); narrow/on-axis radiation cone used. In the centre, the focused beam from (*a*) is placed in red for visual comparison.

At FlexPES there are in total four secondary focal points (two on each branch), and in each point the spot can either be focused or defocused. The spot profiles were characterized at all focal points by using a custom-made YAG screen with a fine-mesh pattern on its surface, in combination with a long-focus microscope. In general, all results match the ray-tracing calculations quite well. As an example, in Fig. 5 ▸ photographs of the beam at the sample position in the first endstation on the SMS branch (EA01 station) are shown for a typical set of beamline parameters (*E* = 265 eV, horizontal exit slit 300 µm, vertical exit slit 40 µm). A result of the corresponding ray-tracing analysis is presented in Fig. 5 ▸(*b*). A similar spot shape with a tail on one side due to mirror-related aberrations can be seen in both measured and calculated profiles. The FWHM for the measured profile is 65 µm (h) × 32 µm (v), which is slightly larger than the predicted size of 52 µm (h) × 28 µm (v), because of the diffraction on the slits, which is not included in the ray-tracing simulations. In practice, the spot shape can be improved (*i.e.* made symmetrical) and the horizontal FWHM can be reduced by closing the baffles in front of the refocusing optics, thus selecting only the central part of the mirror and reducing the aberrations. For example, leaving a horizontal opening of only 1 mm before M4 results in a symmetrical shape and a FWHM below 50 µm, at the cost of 50% intensity loss. As for the vertical spot size, it can be strongly increased by the diffraction effects at low energies and small slits. For example, in going from 30 µm to 10 µm at 150 eV, the vertical FWHM will increase by a factor of four. Therefore, a combination of low energies and small slits is not recommended for the samples where good focusing is critical.

A defocusing of X-rays can be achieved quickly (within a few minutes) by selecting another stripe on M4. The defocused beam intensity at the sample position has a Gaussian distribution in both directions with the FWHM of 1 mm (h) × 0.4 mm (v) (in the case of EA01, but similar also in EB01) for the on-axis radiation [Fig. 5 ▸(*c*)]. Using an off-axis radiation (*i.e.* wider acceptance angles and broader undulator harmonics) can result in a donut-shaped beam profile for certain energies, and should be avoided for the defocused beam arrangement. The defocusing option is very useful when studying radiation-sensitive samples, as the flux density at the spot centre can be reduced by a factor of ∼500 as compared with the standard focused beam without losing the total intensity. At the other focal points, the maximum horizontal sizes of the focused beam spots (FWHM) are: 100 µm (SMS branch, second point), 70 µm (LDM-branch, first point) and 130 µm (LDM-branch, second point); the maximum vertical size is proportional to the vertical slit opening, and varies between 5 and 50 µm in practice (but can become larger at low energies and small slits due to diffraction effects, as mentioned above).

#### 2.3.5. Status of optics

To preserve the surfaces of optical elements clean from carbon contaminations, molecular oxygen can be dosed at several insertion points along the FlexPES beamline. In the presence of the zero-order light, molecular oxygen becomes activated and can remove C contaminations efficiently. An oxygen pressure of the order of 10^−6^ mbar may be needed for several hours to remove severe contaminations. To avoid oxidation of the optical surfaces, we normally use lower but constant O_2_ pressure of the order of 10^−8^ mbar in the M1, PGM and M3 chambers. This keeps the M1, M2 and the grating permanently clean. However, as M3 and M4 are not normally exposed to any zero-order light, they remain contaminated, and need intentional regular cleaning with the zero-order light and O_2_. In practice, we usually have to compromise by using slightly contaminated M3 and M4, with a flux loss of some 20–30% at the C absorption edge around 284 eV.

## 3. Endstations

Below we describe the most essential features of the three endstations located at FlexPES, as at the end of 2022. Further details and updated descriptions can be found at the beamline web page (https://www.maxiv.lu.se/beamlines-accelerators/beamlines/flexpes/).

### 3.1. EA01: PES and NEXAFS spectroscopy in UHV

The permanent endstation EA01 is located at the SMS branch and is designed for studies of electronic properties of surfaces, interfaces, 2D materials and thin films under UHV conditions. The set of techniques includes a combination of XPS, UPS, NEXAFS spectroscopy, resonant PES, secondary electron cut-off measurements and ARPES.

**Figure 6:**
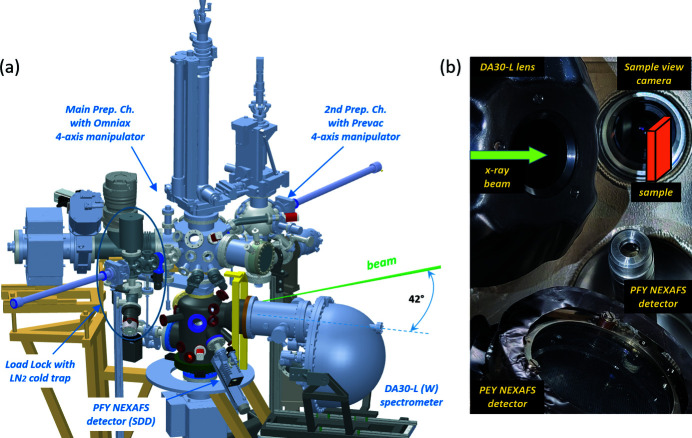
(*a*) 3D CAD-model of the endstation EA01 at the SMS branch; (*b*) arrangement of detectors in the analysis chamber.

The EA01 endstation consists of four main chambers, as shown in Fig. 6 ▸(*a*). The analysis chamber is equipped with the hemispherical analyser DA30-L (from ScientaOmicron) optimized for kinetic energies between 10 and 1500 eV, with a microchannel plate (MCP) stack, a phospho­rous plate, and a CCD camera as a detector. The analyser lens axis is in the horizontal plane, at 42° with respect to the direction of the incident X-ray beam. The entrance slit of the analyser is horizontal, to better match the spot footprint on the sample, especially for a defocused beam. Although most of the time the analyser operates in an angle-integrated mode, it is equipped with the so-called ‘electronic tilt’ option using a deflector lens system. Along with the polar angle rotation on the sample manipulator, the deflector scanning mode enables basic ARPES band mapping with a capability of capturing relatively large segments of the reciprocal space in both directions.

The NEXAFS technique is implemented in several detection modes with different surface/bulk sensitivity, including total, partial and Auger electron yield modes (TEY, PEY and AEY) and partial and total fluorescence yield mode (PFY and TFY). An in-house-built MCP detector with an MCP working diameter of 40 mm is used for collecting electrons in the PEY mode. It is placed right underneath the sample at a small angle from the horizontal plane to increase electron acceptance. This detector is especially useful for highly surface-sensitive XAS measurements. For the photon detection, a PFY energy-dispersive NEXAFS detector (SIRIUS, from Rayspec) is available featuring a silicon-drift detector with relatively large active area of 70 mm^2^, protected by a 70 nm-thick silicon nitride window. It is located in the plane orthogonal to the beam direction. This detector can be effectively used for studying NEXAFS in the bulk and/or in electrically insulating samples. The TEY, PEY, PFY and TFY detection channels are enabled simultaneously in the continuous scanning regime, thus achieving sub-minute acquisition times while preserving high spectral quality. The photograph in Fig. 6 ▸(*b*) illustrates the arrangement of detectors inside the analysis chamber.

The main preparation chamber is placed on top of the analysis chamber. It is linking two other areas – a smaller spherical secondary preparation chamber and a load-lock chamber, used for sample loading to UHV. The primary four-axis manipulator (Omniax, from VACGEN) on top of the main preparation chamber can be used for both measurements and sample treatment. It is equipped with an LHe flow-type cryostat (custom-modified Janis ST-400) allowing for sample cooling down to 20 K (with a special Cu-based manipulator head) or to 40 K (with the standard Mo-based manipulator head). With liquid nitrogen the lowest temperature on sample is 90 K. Samples can be heated with a hot filament (up to 900 K), by electron beam (up to 1400 K), or by direct current through the sample (up to 1500 K). The four-axis manipulator at the second preparation chamber (from Prevac) has the same heating arrangement, but the cooling is possible only with liquid nitrogen. Both preparation chambers have equipment for standard surface science related experiments, such as ports for user-specific devices (evaporators, electrospray sources, UV-lamps, deposition monitors *etc*.), leak valves to introduce gases (some automated, some manual) and ion sputter guns (Prevac IS40-C). The evaporator ports are gated and equipped with individual pumping lines to facilitate using such devices without breaking the vacuum of the respective preparation chambers. The main preparation chamber also has an RGA mass spectrometer (MKS Microvision2), a quartz thickness monitor (Prevac TMC13) and a LEED optics (BDL800 from OCI). In total, up to 18 sample plates can be stored in the system: six in the load-lock and six in each preparation chamber. Up to six gases or vapours can be introduced from the gas cabinet via dedicated gas lines.

If it is desirable to correlate the X-ray spectroscopy data with the surface morphology, samples can be transferred in UHV from/to the MAX IV STM Support Lab using a dedicated vacuum suitcase.

### 3.2. EB01 (ICE): multi-coincidence momentum imaging on gas phase targets

The ICE (‘Ions in Coincidence with Electrons’) endstation is a mobile coincidence spectroscopy setup equipped with a REMI used for high-resolution measurements of the three-dimensional momentum distributions of electrons and ions. With its ability to detect a number of particles in coincidence, the REMI is particularly suitable to the investigation of photo-induced molecular processes/fragmentation dynamics of molecules and clusters.

The COLTRIMS REMI (from RoentDek GmbH) is adjustable in length and electrostatic field geometry – affording some technical flexibility and therefore a broad scientific applicability. With the possibility to adjust the applied fields, the instrument can also be used during both single-bunch and multi-bunch delivery. The REMI comprises two time-of-flight (ToF) spectrometers (electron and ion) mounted opposite to each other on opposite sides of an ‘interaction region’. Each spectrometer consists of a number of open copper electrodes, which can be connected in various ways in order to realize different electrostatic field configurations. The REMI is equipped with two HEX100-75 delay line/MCP detector arrangements, *i.e.* three-layer delay line detectors (diameter 100 mm) and MCPs of 75 mm diameter.

The main vacuum chamber is a multi-port CFEL-ASG multipurpose (CAMP) chamber (Strüder *et al.*, 2010 ▸) designed for experiments on volatile targets involving imaging/photoexcitation techniques. The vacuum chamber extensions are custom designed to ensure flexibility in their assembly (*i.e.* they can be repositioned/removed depending on the chosen length of the ion and electron spectrometers) and the strategically placed windows aid safe mounting of the detectors.

A complete description of this setup and its performance will be given in a separate publication (Walsh *et al.*, 2023 ▸).

### 3.3. EB02: XPS on LDM and non-UHV targets

The permanent endstation EB02 is located at the LDM-branch; it was originally designed for electron spectroscopy studies on both gas-phase and substrate-supported samples (Svensson *et al.*, 1996 ▸), and was previously used at the I411 beamline of the MAX Laboratory. It has three main chambers: an analysis chamber, a preparation chamber and an introduction chamber. The analysis chamber is equipped with a Scienta R4000 electron energy analyser and can be rotated together with the analyser around the photon beam direction without breaking vacuum. In this way, the angle between the analyser and the photon polarization plane can be varied on demand. The analyser lens axis is at 90° to the beam direction, and its entrance slit is parallel to the beam direction.

Multiple ports of up to CF160 size facilitate mounting of modular setups on the analysis chamber for *in situ* delivery of LDM samples. The preparation chamber has multiple ports for mounting an RGA mass spectrometer (Hiden Analytical HAL301), an ion sputter gun (Prevac IS40-C), gas dosing valves and user-specific evaporators. Solid samples can be transferred via an introduction chamber equipped with a storage carousel for eight samples. A dedicated mobile glove box can be docked to the load-lock chamber for air-free insertion of moisture-sensitive samples. Solid samples can be transferred to the analysis chamber with a four-axis manipulator (Omniax, from VACGEN) attached to the preparation chamber along the light axis. The Mo-based sample receiver can accept standard flag-type sample plates, which are positioned at 45° to the beam. User-specific sample heads can replace the standard receiver if necessary.

For XPS studies on LDM samples, there are at present four standalone sample delivery systems. For experiments on gaseous substances and vapours, there is an in-house built, flange-mounted gas cell capable of creating a dense gas/vapour flow through a closed compartment with a small entrance hole for the incoming X-rays. The cell is equipped with facilities for *in situ* alignment.

**Figure 7:**
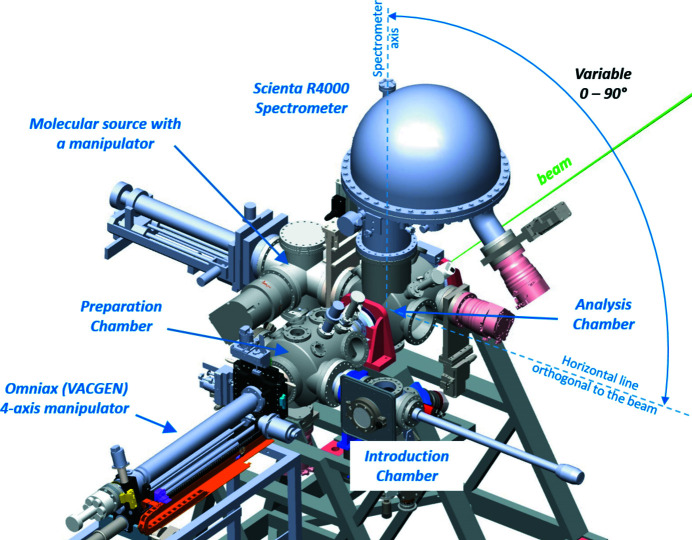
3D CAD-model of the endstation EB02 at the LDM-branch.

Another setup is the molecular/cluster beam apparatus, which can generate dense beams of atoms, molecules and free clusters from gases and vapours of liquids. It is based on gas adiabatic expansion and is equipped with two turbo-molecular pumps for efficient differential pumping. When attached to a CF160 port of the analysis chamber (Fig. 7 ▸), the setup is separated from the ionization volume via a skimmer with a 0.3–0.5 mm opening. To handle the gas load, either an additional turbo-pump or a liquid-nitro­gen cold trap is installed opposite to the sample flux.

The third sample-generating apparatus is the liquid-jet setup producing a microjet of liquid, and thus enabling electron spectroscopy studies into the chemistry of solutions. The liquid is pushed through a cylindrical quartz nozzle (opening typically 20–25 µm in diameter, from Advanced Microfluidic Systems GmbH) into vacuum, using standard high-performance liquid chromatography (HPLC) equipment. Aqueous samples are the most common; other solvents are possible, though limited by the vapour pressure. To handle the substantial vapour load during the operation, the liquid jet is isolated via a differentially pumped compartment, with a small opening towards the spectrometer lens at the tip of a conical skimmer. After crossing the X-rays, the jet is captured in a liquid-nitro­gen-cooled trap mounted on the opposite side of the analysis chamber.

The fourth sample-delivery system is a cluster source, which can produce a beam of nanoscale particles of metals, oxides, nitrides, hydrides and sulfides from solid targets by magnetron sputtering. The experimental arrangement involves dedicated equipment for differential pumping separating the source from the analysis chamber by a skimmer with an opening of 1–2 mm positioned in front of the ionization point. An additional turbo-molecular pump is usually necessary and can be mounted directly on the analysis chamber below the beam.

## 4. Control system

The motions of all optical elements, masks, baffles, slits and diagnostic tools are motorized and controlled with high precision for convenient and fast alignment of the optics according to the specific user demands. Several cameras are used to monitor beam images on fluorescent screens (located on beam-defining apertures and exit slits). All these beamline elements can be accessed from a synoptic: a graphical user interface (GUI) providing an overview of the entire beamline. It also includes elements to access macros, *e.g.* for switching between the branches, exchange gratings or for switching the M4 mirrors between focused and defocused settings.

As for almost all beamlines at MAX IV, the control and data acquisition system at FlexPES is built in the open-source software suite Tango (Tango, 2022 ▸) and uses a Python-based Sardana framework (Coutinho *et al.*, 2011 ▸) to communicate with the IcePAP motor controllers (Janvier *et al.*, 2013 ▸). All low-level devices (vacuum gauges, pumps, valves, *etc.*) as well as various safety-related and instrument-protecting interlocks are controlled via the PLC (programmable logic controller) system. All these devices and many of the high-level instruments (all motors and some detectors) can be controlled via their Tango device servers, which allows them to be easily integrated into various GUIs.

Similarly to the beamline, most elements on the EA01 and EB02 endstations, *e.g.* vacuum systems, manipulator axes, cameras, sample heating, ion sputtering, gas inlet valves, *etc*., are included in the control system, motorized (where applicable), and accessible via GUIs. The strong control system integration provides convenient and time-saving software tools, *e.g.* for the sputter-annealing sample preparation cycles or for the fast sequential NEXAFS measurements (with varying energy regions, *c*_ff_ values of the PGM, sample positions, detector settings, slit settings, *etc*.). It also facilitates remote user support and troubleshooting of the experimental issues by the staff, and enables a limited remote user access to the measurement process. Finally, a project for integrating the photoemission data acquisition into the Tango/Sardana control system is ongoing.

## 5. Summary

The FlexPES beamline at the 1.5 GeV ring of the Swedish National Synchrotron Radiation Laboratory MAX IV is a versatile experimental facility allowing researchers to study the structure and dynamics at solid surfaces and interfaces, in liquids, gas-phase molecules and free clusters. The main experimental methods are high-resolution photoelectron spectroscopy, fast X-ray absorption spectroscopy and electron–ion/ion–ion coincidence techniques, at high photon flux and over a wide range of photon energies (40–1500 eV). The three current endstations offer users not only efficient and informative channels of acquisition but also a number of *in situ* sample-generation possibilities creating samples and environments relevant for catalysis, environmental chemistry, fabrication of novel materials and devices, micro-chemistry of solutions, *etc*. A high degree of automation and motorization facilitates a correspondingly high degree of experimental reproducibility, ease of control, accuracy and efficiency.

## Figures and Tables

**Table 1:** Characteristics of plane gratings installed at FlexPES

	Groove density (lines mm^−1^)	Optical area (mm)	Substrate	Coating	Slope errors (arcsec)	Blaze angle (°)
PG1	1221	110 × 30	Si	Au	0.1	1.35
PG2	400	110 × 30	Si	Au	0.04	1.55

**Table 2:** Mirror parameters of the FlexPES beamline

	M1	M2	M3a / M3b	M4a_1_ / M4a_2_	M4b_1_ / M4b_2_
Shape	Cylindrical	Plane	Toroidal / toroidal	Toroidal / toroidal	Toroidal / toroidal
Deflection	Horizontal	Vertical	Horizontal / Horizontal	Horizontal / Horizontal	Horizontal / Horizontal
Distance (m)	15	Variable	19	32.5 / 32.5	28 / 28
Incidence angle (°)	2	1–13	2	2 / 2	2 / 2
Block size (mm)	300 × 40 × 60	640 × 40 × 110	260 × 40 × 40 / 260 × 40 × 40	260 × 75 × 40	260 × 75 × 40
Optical area (mm)	280 × 20	640 × 40	240 × 20	Two stripes of 240 × 20	Two stripes of 240 × 20
Substrate material	Si	Si	Si	ZERODUR	ZERODUR
Coating material	Au	Au	Au	Au / Au	Au / Au
Roughness (Å)	3	3	3	5 / 5	5 / 5
RMS slope error (tangential / sagittal) (arcsec)	0.08 / <1	0.1 / 0.1	0.23 / <1	0.19 / <1	0.23 / <1
			0.13 / <1	0.14 / <1	0.26 / <1
RMS radii (tangential / sagittal) (mm)	∞ / 1047	∞ / ∞	271278 / 698	107935 / 102	76410 / 70
			238740 / 489	175101 / 143	114615 / 93

**Table 3:** Parameters for the chopper installed at the FlexPES LDM-branch

Disc diameter (mm)	Fixed slit width (µm)	Disc slit width (µm)	Number of slits	Rotation frequency range (Hz)	Period (µs)
240	85	85	120	788.6–394.3	10.57–21.13
